# Opioid weaning and pain management in postsurgical patients at the Toronto General Hospital Transitional Pain Service

**DOI:** 10.1080/24740527.2018.1501669

**Published:** 2018-08-20

**Authors:** Hance Clarke, Saam Azargive, Janice Montbriand, Judith Nicholls, Ainsley Sutherland, Liliya Valeeva, Sherif Boulis, Kayla McMillan, Salima S. J. Ladak, Karim Ladha, Rita Katznelson, Karen McRae, Diana Tamir, Sheldon Lyn, Alexander Huang, Aliza Weinrib, Joel Katz

**Affiliations:** a Pain Research Unit, Department of Anesthesia and Pain Management, Toronto General Hospital, University Health Network, Toronto, Canada; b Department of Anesthesia, University of Toronto, Toronto, Canada; c Queen’s School of Medicine, Faculty of Health Sciences, Queen’s University, Kingston, Canada; d Department of Anaesthesia and Intensive Care, Queen Elizabeth Hospital, Bridgetown, Barbados; e Cave Hill Campus, University of the West Indies, Wanstead, Barbados; f Department of Anesthesiology, St. Paul’s Hospital, Providence Health Care, Vancouver, Canada; g Department of Anesthesia and Pain Management, Toronto General Hospital, University Health Network, Toronto, Canada; h University of Toronto, Toronto, Canada; i Transitional Pain Service, Department of Anesthesia and Pain Management, Toronto General Hospital, University Health Network, Toronto, Canada; j Canada Research Chair in Health Psychology, Department of Psychology, York University, Toronto, Canada

**Keywords:** Chronic pain, opioids, postoperative pain management

## Abstract

**Background:**

The perioperative period provides a critical window to address opioid use, particularly in patients with a history of chronic pain and presurgical opioid use. The Toronto General Hospital Transitional Pain Service (TPS) was developed to address the issues of pain and opioid use after surgery.

**Aims:**

To provide program evaluation results from the TPS at the Toronto General Hospital highlighting opioid weaning rates and pain management of opioid-naïve and opioid-experienced surgical patients.

**Methods:**

Two hundred fifty-one high-risk TPS patients were dichotomized preoperatively as opioid naïve or opioid experienced. Outcomes included pain, opioid consumption, weaning rates, and psychosocial/medical comorbidities.

**Results:**

Six months postoperatively, pain and function were significantly improved. Opioid-naïve and opioid-experienced patients reduced consumption by 69% and 44%, respectively. Forty-six percent and 26% weaned completely. Consumption at hospital discharge predicted weaning in opioid-naïve patients. Pain catastrophizing, neuropathy, and recreational drug use predicted weaning in opioid-experienced patients.

**Conclusions:**

The TPS enabled almost half of opioid-naïve patients and one in four opioid-experienced patients to wean. The TPS successfully targets perioperative opioid use in complex pain patients.

## RÉSUMÉ

**Contexte**: La période périopératoire constitue un créneau déterminant pour s’attaquer à la consommation d’opioïdes, en particulier chez les patients qui ont une histoire de douleur chronique et de consommation préopératoire d’opioïdes. Le Service de la douleur transitionnelle de l’Hôpital général de Toronto a été mis sur pied pour s’attaquer au problème de la douleur et de la consommation d’opioïdes après une chirurgie.

**But**: Présenter les résultats de l’évaluation du programme du Service de la douleur transitionnelle à l’Hôpital général de Toronto en mettant l’accent sur les taux de sevrage des opioïdes ainsi que sur la prise en charge de la douleur chez les patients n’ayant jamais consommé d’opioïdes et ceux qui en avaient déjà consommé.

**Méthodes**: Avant d’être opérés, 251 patients à haut risque du Service de la douleur transitionnelle ont été séparés en deux groupes, l’un réunissant les patients n’ayant jamais consommé d’opioïdes et l’autres réunissant ceux qui en avaient déjà consommé. Les résultats portaient sur la douleur, la consommation d’opiodes, les taux de sevrage, ainsi que les comorbidités psychosociales et médicales.

**Résultats**: Six mois après l’opération, la douleur et le fonctionnement s’étaient améliorés de manière significative. Les patients qui n’avaient jamais consommé d’opioïdes et ceux qui en avaient déjà consommé avaient réduit leur consommation de 69 % et 44 % respectivement, et 46% et 26 % d’entre eux étaient complètement sevrés. La consommation au moment du congé de l’hôpital prédisait le sevrage chez les patients qui n’avaient jamais consommé d’opioïdes auparavant. La catastrophisation de la douleur, la neuropathie et l’usage de drogues récréatives prédisaient le sevrage chez les patients qui avaient déjà consommé des opioïdes.

**Conclusions**: Le Service de la douleur transitionnelle a permis le sevrage de près de la moitié des patients qui n’avaient jamais comsommé d’opioïdes auparavant et à un patient sur quatre parmi ceux qui avaient déjà consommé des opoïdes auparavant. Le Service de la douleur transitionnelle cible avec succès la consommation préopératoire d’opioïdes chez les patients souffrant de douleur complexe.

## Introduction

### Complex postsurgical pain

Poorly managed postsurgical pain (PSP) can interfere with recovery and rehabilitation, increasing the length of admission and compromising quality of life for patients.^1^ Inadequate acute postsurgical pain management can lead to the development of chronic postsurgical pain (CPSP).^2^ Severe disability as a consequence of CPSP has been estimated to affect 5%–10% of patients one year following major surgery.^3^ Incidence rates for CPSP have been reported to be as high as 50% to 70% one year after amputation or thoracic surgery.^4^

In the province of Ontario, Canada, 49.2% of major surgery patients who were not using an opioid before surgery are discharged from hospital with an opioid prescription, and 3.1% continue to use opioids 90 days after surgery.^5^ This evidence comes from patients in the general population who were not taking opioids prior to surgery, making 3.1% an underestimate of persistent opioid use rates in patients who do take opioids before surgery and who are at high risk of developing CPSP. The risk factors associated with the development of CPSP have been previously elucidated and are described in Table 1.^2^

**Table 1.** Referral criteria for the TPS.^a^

1.	“Pain alert” patients
	Presurgical chronic pain
	History of drug abuse
	Currently on opioid, methadone, or buprenorphine maintenance therapy
2.	Severe postsurgical pain
	Prolonged APS stay
	Surgical patients with repeat APS consultation
	Medically stable postsurgical patients with complex pain problems that prevent discharge
3.	High postsurgical opioid consumption
	Consumption of >90 MEQ/day
	Methadone or buprenorphine patients without community pain specialist
	Patients discharged with a prescription for long-acting opioid
	Interventional postsurgical procedures (e.g., stump catheters postamputation)
4.	Emotional distress
	Anxiety and/or depression diagnosed by a mental health professional
	High level of pain catastrophizing
	Other psychosocial concern identified by APS or TPS questionnaires

^a^Adapted with permission from Katz et al.^13^

TPS = Transitional Pain Service; APS = Acute Pain Service; MEQ = morphine equivalents.

Opioids continue to be the most effective option for the management of acute pain both in posttraumatic and postsurgical settings.^6^ However, concerns about the current opioid crisis and the risk of persistent opioid use after surgery have highlighted the need for improved perioperative and postdischarge care.^7^ Several authors have called for more refined interventions to reduce opioid use and the escalation of opioid doses after surgery, including the implementation of a Transitional Pain Service (TPS) as a practical model to address the multiple factors associated with the transition from hospital to community for complex postsurgical pain patients.^8–12^

### The Toronto General Hospital Transitional Pain Service

Launched in 2014, the TPS at Toronto General Hospital (TGH) is, to our knowledge, the first perioperative pain service to comprehensively address the specific problems of CPSP and opioid use at three perioperative stages: (1) preoperatively, (2) postoperatively in hospital, and (3) postoperatively in an outpatient setting for up to 6 months after surgery. This pain service aims to bridge the gap in suboptimal pain management for complex patients and in unsafe prescribing practices. Toronto General Hospital performs 6000 surgeries per year; 4000 are considered major surgery (thoracic, cardiac, urological, general, gynecological, and otolaryngology), making it an ideal institution within which to pilot and implement a service such as the TPS.^13^ The potential cost savings to the institution and the health care system as a whole has been described previously.^14^ The structure and associated protocols of the TPS, including descriptions of recruitment, risk factors, and interventional approaches, are discussed in previous publications in detail.^14^

Participation by the patients described in this article continued for an average of 6 months after surgery. Patients who were at high risk for developing pain and problematic opioid use were targeted in order to safely and efficiently wean them from opioids and optimally manage their pain.^5,12^ This population is not only at higher risk for misusing opioids but are slower to taper and require additional care when weaning.^13^ One critical aspect to the pain management approach at the TPS involves obtaining the patients’ commitment to wean from their opioid-based medication with the aid of nonpharmacologic and nonopioid medication strategies, making an interdisciplinary team with multimodal interventions necessary.

Briefly, the interdisciplinary team charged with treating patients at the TPS includes nurse practitioners, physicians, psychologists, and other allied health professionals.^13^ Nurse practitioners and physicians specializing in anesthesia, pain management, and addiction are responsible for determining and managing each patient’s care plan and applying pharmacological approaches to pain, prioritizing alternatives to opioids whenever possible.^14,15^ The clinical psychologists make up a large proportion of the team, providing behavioral interventions for pain and addiction as part of their multimodal approach.^16^ Physiotherapists and other allied health professionals offer other services based on patient need.

Broadly, the goals of the TPS are to effectively manage long-term pain, maintain function, reduce opioid consumption, and monitor the efficacy of these interventions. The purpose of this article is to report the preliminary results of the TPS focusing on quantifying opioid consumption at the time of discharge following major surgery in opioid-naïve and opioid-experienced patients. This article will also describe the relationship between opioid consumption and psychometric parameters related to pain to evaluate the psychosocial variables that should be targeted as predictors of opioid weaning.

## Methods

This study was approved by the research ethics board of TGH (University Health Network, Toronto, ON, Canada: REB#14–7705AE) and by the Human Participants Review Sub-Committee York University (based on UHN approval: 14-7705-AE). All data included in this article were anonymized from patients who gave written informed consent and were free to withdraw their consent at any time.

### Eligibility and exclusion criteria

All patients were required to be 18 years of age or older at the time of recruitment. TPS eligibility was determined during the pre- and postoperative periods. Patients were referred to the TPS by physicians who identified them as being high risk for developing chronic pain. We have also attempted to target those with the most protracted weaning rates, modifying our approach and expectations regarding their expected progress through the weaning process.^13^ Our team has developed risk factors for developing chronic pain postsurgically, which were used as a guide for physicians at TGH (Table 1).^2^ These were not used as specific referral criteria but rather as a way to educate members of the health care team to better identify patients with problematic coping skills. These risk factors included the following:

1. History of chronic pain, drug abuse, current or previous opioid therapy, on methadone or buprenorphine).
2. Patients followed by the Acute Pain Service (APS) for more than 3 days postoperatively for poorly controlled postoperative pain (average pain score > 4 on an 11-point scale) or complex pain patients requiring multiple pain consultations prior to hospital discharge after the APS had signed off.
3. Patients consuming more than 90 mg/day of oral morphine equivalents (MEQ) on postoperative day 1, patients being discharged on long-acting opioids, patients on buprenorphine or methadone without community follow-up, or those requiring specialized postsurgical interventional procedures.
4. Patients with psychological risk factors for persistent opioid use or drug addiction such as a past or present history of anxiety, depression, and high pain catastrophizing scores, identified by the APS or TPS.

### Patients

In total, 411 patients were seen by the TPS between May 2014 and July 2017. Consent for participation was obtained from 304 surgical patients. Of those who agreed to participate, 53 were missing information on opioid consumption, leaving 251 patients (111 female [44.4%], mean age = 50.88 years, SD = 14.01, range = 19–81 years) for analysis. Patients were assessed through three referral routes at different time points: (1) patients arriving to hospital for surgery (*n* = 51, 20%); (2) admitted patients seen by the acute pain service (*n* = 119, 47%); and (3) patients seen postsurgically at the TPS clinic after hospital discharge (*n* = 81, 32%). Surgery types included thoracic (27%), general (16%), otolaryngology (12%), transplant (11.2%), cardiac (10%), vascular (8%), obstetrics and gynecology (4.4%), plastics (4.4%), urology (4.4%), neurosurgery (1.2%), orthopedics (0.4), and other (0.8%).

### Design

This is a single-center observational study of the TPS that followed patients to determine opioid use and weaning rates. Data are reported from three time points: at admission to hospital (presurgical); after hospital discharge (postsurgical); and at a mean of 6 months following surgery. Patient information on physical and psychological functioning was collected during TPS visits by self-report and medical history with prespecified forms and entered into the database.

Exclusion criteria were limited to those based on publication of data: (1) Patient refusal to have their data used for publication purposes or (2) no signed consent form.

### Interventions within the TPS

Broadly, the TPS interventions fall under three main categories in the context of an interdisciplinary framework: nonpharmacological and pharmacological pain management, opioid weaning, and psychosocial/psychological interventions. An overview including the development and structure of the TPS has been previously described.^17,18^ Posthospital discharge visits occurred twice a month for the first 2 months after surgery typically followed by monthly visits thereafter or tailored to the specific needs of the patient.

Pharmacological interventions to manage pain included opioid and nonopioid analgesics. Nonopioid medications included acetaminophen, nonsteroidal anti-inflammatory drugs, and anticonvulsants (i.e., gabapentinoids). Patients are engaged early and involved in the decision-making process when determining the most suitable weaning strategies, including nonpharmacological pain management techniques and psychological intervention where appropriate. Methadone and buprenorphine/naloxone protocols are applied for patients for whom typical opioids no longer meet their analgesic needs. Because of their analgesic properties in addition to their use as opioid replacement and addiction therapy, a rotation to buprenorphine/naloxone was used to balance analgesia with improved functioning and quality of life after years of chronic pain.

Psychological treatment to help with opioid weaning and pain management and/or to address concomitant mental health problems is provided by a registered clinical psychologist and/or several supervised clinical psychology graduate student trainees or registered clinical psychology postdoctoral fellows under supervised practice. The most used psychological intervention at the TPS is acceptance and commitment therapy (ACT), a form of cognitive–behavioral therapy tailored to chronic pain. The ACT intervention targets the management of inner experiences (e.g., pain) and the nurturing of adaptive psychosocial behaviors by employing mindfulness-based approaches and a visual aide known as the ACT Matrix.^17,18^

### Database variables

The variables collected include (1) patient demographics, (2) intraoperative and postoperative parameters, and (3) psychometrics for pain management, opioid consumption, and psychosocial indices as follows.

1. Patient demographics and medical information variables include age, gender, ethnicity, previous medical history, family history, chronic pain history, current problem list, and surgical procedure. Preoperative medication included opioids, anticonvulsants, nonsteroidal anti-inflammatories, acetaminophen, acetylsalicylic acid (ASA), angiotensin-converting enzyme inhibitors, calcium channel blockers, fluids, statins, beta blockers, steroids, and antidepressants.
2. Intraoperative and postoperative variables include date of surgery, type of surgery, surgeon, anesthesiologist, type of postoperative analgesia, analgesic medication, duration of analgesia, and pain scores via visual analogue scale/numeric rating scale (NRS). Physiotherapy outcomes include activity and sleep.
3. Psychometric variables were those for monitoring psychosocial functioning, subjective pain experience, and those specific for TPS psychologists: The Brief Pain Inventory–Short Form (BPI-SF), the Hospital Anxiety and Depression Scale (HADS), the Pain Catastrophizing Scale (PCS), and the McGill Pain Questionnaire–Short Form 2 (SF-MPQ-2).

### Psychometric questionnaires

Psychosocial measures were assessed at various timepoints, depending on the patient’s clinical progress, to evaluate the patient’s initial and ongoing level of psychosocial functioning, to develop a clinical plan for psychological treatments, and to monitor ongoing progress. Psychosocial assessments were completed as deemed necessary by the clinical psychologists involved in the TPS.

#### Brief Pain Inventory–Short Form

The BPI-SF^19^ is one of the most widely used scales for measuring pain and pain interference in patients with a variety of chronic pain problems. The BPI-SF is a 16-item, self-report questionnaire that consists of a body diagram patients use to mark the location of their pain, a question about pain treatments and medications, and one concerning the percentage of relief obtained. The BPI-SF uses an 11-point NRS (0–10) with end points labeled *no pain* and *pain as bad as you can imagine* to measure the intensity/severity of the “worst,” “least,” “average,” and “now” (present) pain. Another 0–10 NRS (with end points labeled *does not interfere*” and *completely interferes*) is used to measure the impact of pain on functioning (interference) in seven daily activities, including general activity, mood, walking ability, work, relations with other people, sleep, and enjoyment of life. The BPI has been used extensively in a variety of pain conditions and has been shown to have excellent psychometric properties, including validity and reliability in patients with chronic nonmalignant pain.^20^

#### Pain Catastrophizing Scale

The PCS^21^ consists of 13 items describing thoughts and feelings that individuals may experience when they are in pain. Each item is rated on a 5-point scale ranging from *not at all* (0) to *all the time* (4). The PCS yields a total score and three subscale scores assessing (1) rumination, (2) magnification, and (3) helplessness. The PCS has good to excellent psychometric properties.^21^

#### Hospital Anxiety And Depression Scale

The HADS^22^ is the most widely used scale for measuring symptoms of anxiety and depression among medical inpatients, outpatients and the general population and consists of seven anxiety and seven depression-related items. The psychometric properties (test–retest reliability, internal consistency, construct, and concurrent validity) of the HADS are excellent.^22^

#### Mcgill Pain Questionnaire–Short Form 2

The SF-MPQ-2^23^ is a 22-item, expanded and revised version of the SF-MPQ designed to measure the qualities of neuropathic and nonneuropathic pain. Exploratory and confirmatory factor analyses revealed the presence of the following four factors or subscales: (1) continuous pain, (2) intermittent pain, (3) neuropathic pain, and (4) affective pain descriptor. Preliminary analyses indicate that the SF-MPQ-2 has very good to excellent psychometric properties.^23^

### Outcomes

#### Primary outcomes

- Duration of opioid therapy posthospital discharge.
- Rates of opioid weaning for opioid-naïve (% and MEQ postoperative [discharge] and 6 months) and opioid-experienced patients (% and MEQ pre-operative, postoperative [discharge], and 6 months).

#### Secondary outcomes

- Pain and pain interference (hospital discharge and 6 months after surgery).
- Predictors of opioid weaning and pain measures.
- Psychosocial function for opioid-naïve and opioid-experienced patients (% between first and last TPS visit).

### Data analysis

Patients were dichotomized into two groups based on their presurgical opioid consumption. Opioid-experienced patients were those consuming >0 MEQ/day. All other patients with 0 MEQ/day presurgically were classified as opioid naïve. Although medical histories were reviewed to supplement our quantification of past opioid consumption, data on this variable were limited to self-report. Opioid replacement therapies (buprenorphine/naloxone) were not included in the calculation of daily MEQ dose. Outliers in opioid consumption who differed by a standardized residual change score of greater than four were removed from the sample for analysis.

Means and standard deviations were used to report continuous variables. Percentages and frequencies were used to describe nominal or categorical variables. Continuous demographic variables, nonopioid and recreational drug consumption, and medical comorbidities were compared between opioid-naïve and opioid-experienced groups using *t* tests and nominal or categorical variables were compared with chi-squared tests. Analysis of covariance (ANCOVA) was computed to compare opioid consumption and weaning rates between and within groups using baseline opioid consumption as a covariate. Pain and function were also analyzed with ANCOVAs within and between groups with baseline NRS pain and pain interference scores as covariates. Pearson correlation coefficients were computed between opioid dose, pain outcomes, and psychometric scales of interest. All data analyses were conducted using SPSS version 23.

## Results

### Group characteristics

Table 2 shows demographic information for the two groups (opioid-naïve = 112, opioid-experienced = 139). The percentage of females in the opioid-naïve group (55%) was significantly greater than that in the opioid-experienced group (35%; *P* = 0.003). The mean number of months since surgery for the whole study sample was 6 months and 5.24 (SD = 5.7) and 6.61 (SD = 6.59) for the opioid-naïve and opioid-experienced groups, respectively (not significant). The mean number of visits to the TPS was 5.67 (SD = 7.5) and 5.82 (SD = 6.8) for the opioid-naïve and opioid-experienced groups, respectively (not significant). Preoperatively, opioid-experienced patients had significantly higher rates of medical comorbidities, including diabetes (*P* = 0.001), peptic ulcer disease (*P* = 0.002), gastroesophageal reflux disease (*P* = 0.004), and chronic pain (*P* = 0.0004).

**Table 2.** Demographic variables and statistical comparison between groups.

	Opioid naïve	Opioid experienced	Significance level
Demographic	(*n* = 112)	(*n* = 139)	(*P*)^a^
Age (mean± SD)	49.0 (15.0)	52.4 (14.0)	0.063
Female	61 (55%)	50 (36%)	0.003*
Mean # of TPS visits (SD)	5.7 (7.5)	5.8 (6.8)	0.187
Recreational drug use^b^	16.2%	25.9%	0.07
Alcohol dependence	12.5%	17.3%	0.35
Smoking status			0.3
Past	45.3%	40.5%	
Current	19.8%	29.4%	
Comorbidities			
Chronic pain	35.8%	81.7%	0.0004**
Pulmonary disease	25.6%	22.2%	0.574
Heart failure	4.7%	7.9%	0.41
Diabetes	12.6%	21.6%	0.001*
Peptic ulcer disease	3.6%	8.6%	0.002*
GERD	34.2%	45.3%	0.004*

^a^Significance levels were compared using chi square with the exception of “Age” and “# of TPS visits” (*t* test).

^b^Recreational drug use was self-reported nonprescription use of cannabis, cocaine, LSD, gabapentin, or unspecified.

*P < 0.005. **P < 0.0005.

TPS = Transitional Pain Service; GERD = gastroesophageal reflux disease.

#### Opioid consumption and weaning rates

Table 3 shows opioid consumption and weaning rates for opioid-naïve and opioid-experienced patients. Two outliers were removed from each group based on opioid consumption. Opioid-naïve patients (*n* = 110) were taking a mean of 106.7 ± 80.6 MEQ at the time of hospital discharge. Daily opioid consumption was reduced to a mean of 37.3 ± 61.1 MEQ/day at the final TPS visit (65% decrease) an average of 6.6 months after surgery. Opioid-experienced patients (*n* = 137) were taking a mean of 78.8 ± 100.2 MEQ/day prior to surgery. At the time of hospital discharge, these patients were consuming an average of 140.5 ± 124.0 MEQ/day, which was reduced to 78.3 ± 113.9 MEQ/day by their last TPS visit (44.3% decrease) an average of 5.2 months after surgery. ANCOVA, using MEQ at the time of discharge as a covariate, revealed that the MEQ dose at the final TPS visit was significantly higher for the opioid-experienced versus opioid-naive group (F = 8.256, *P* = 0.004, η^2^*_p_* = 0.032).

**Table 3.** Summary of opioid consumption and weaning rates.

Measures	Opioid naïve	Opioid experienced
	(*n* = 110) †	(*n* = 137)^a^
Mean MEQ (mg) consumed (mean ± SD)		
Presurgical	0	78.8 (100.2)
Hospital discharge (postsurgical)	106.7 (80.6)	140.5 (124.0)
Final TPS visit	37.3 (61.1)	78.3 (113.9)
% Decreased from discharge	69.4%	44.3%
Weaning rate achieved^b^		
No longer taking an opioid (100%)	49 (44.5%)	35 (25.6%)
Reduced to ≥50%	39 (35.5%)	48 (35.0%)
Reduced to <50%	11 (10.0%)	28 (20.4%)
Increased from hospital discharge	11 (10.0%)	26 (19.0%)

^a^Two outliers from each group, with a standardized residual change score of greater than four, were removed for a total of four outliers.

^b^Weaning rates are measured from hospital discharge to a mean time of 6 months.

MEQ = morphine equivalents; TPS = Transitional Pain Service.

**Figure 1.**
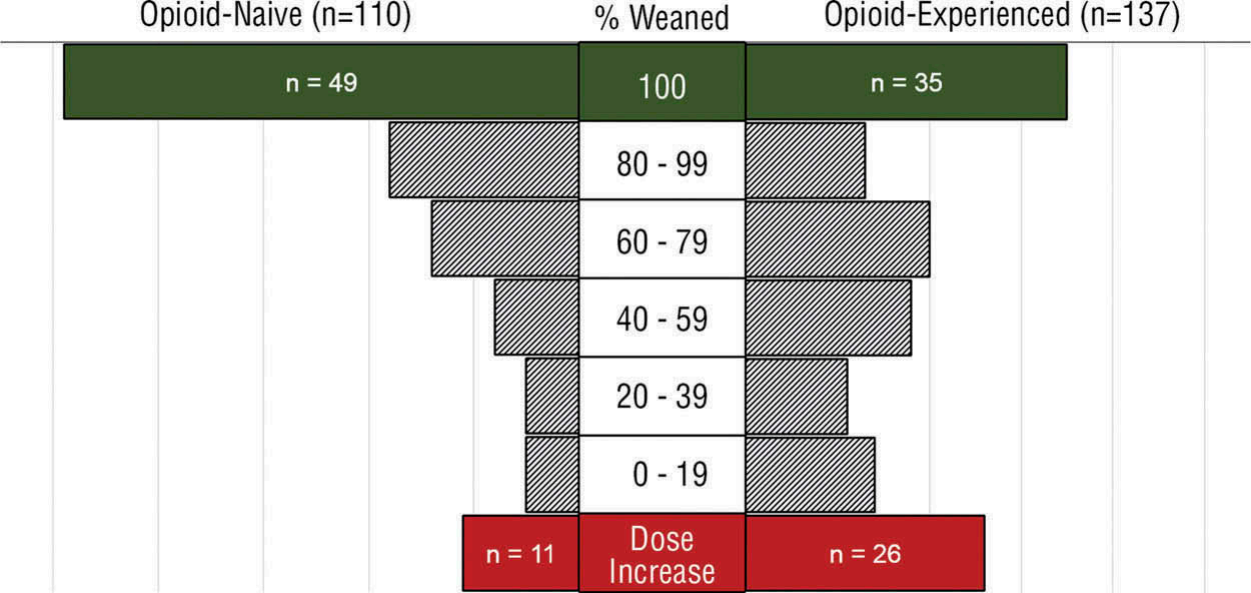
Frequency of weaning, MEQ dose reduction, or MEQ increase 6 months after surgery arranged by percentage change in opioid use since hospital discharge in opioid-naïve patients and opioid-experienced patients. Note that of the 49 opioid-naïve and 35 opioid-experienced patients who had been completely weaned from opioids, 1 and 3 were taking buprenorphine/naloxone, respectively.

Six months after surgery, 49 opioid-naïve patients (44.5%) had been successfully weaned to 0 MEQ/day, 39 (35.5%) had reduced their opioid use by ≥50% of their discharge dose, 11 (10%) had reduced their opioid use by <50%, and 11 (10%) had increased their opioid use since hospital discharge (Figure 1). Six months after surgery, 35 opioid-experienced patients (25.6%) had been successfully weaned, 48 (35%) had reduced their opioid use by ≥50% of their discharge dose, 28 (20.4%) had reduced their opioid use by <50%, and 26 (19%) had increased their opioid use since hospital discharge (Figure 1).

The subgroup of opioid-experienced patients who *increased* their opioid consumption after discharge represents a population of interest who faced the most challenge weaning from opioids. When comparing this subgroup to the remainder of opioid-experienced patients who reduced opioid consumption, a greater proportion were male (21/26; χ^2^ = 4.432, *P* = 0.035). This subgroup was also more likely to have been diagnosed with a mental health disorder (χ^2^ = 5.822, *P* = 0.044) when compared to the opioid-experienced patients who had reduced their opioid consumption. Lastly, organ transplant patients were overrepresented in this group compared to the subgroup that decreased opioid consumption (χ^2^ = 4.151, *P* = 0.042).

### Predictors of opioid consumption and weaning rates

**Figure 2.**
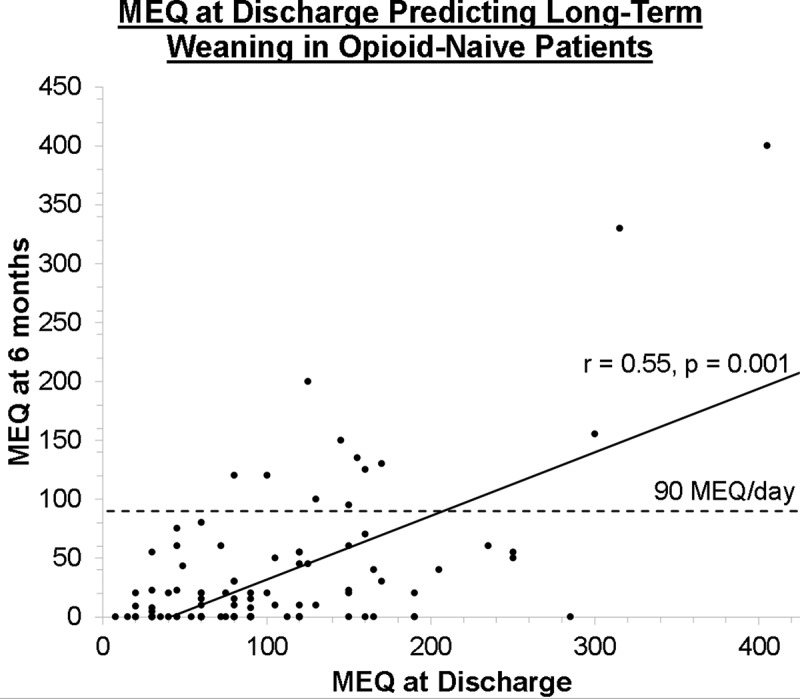
Graph showing the relationship between MEQ dose after discharge and the final recorded MEQ dose 6 months after surgery.

MEQ at hospital discharge predicted percentage reduction in opioid consumption from discharge to the final TPS visit (*r* = 0.23, *P* = 0.02) among all patients (*n* = 251). These reductions were also predicted by baseline levels of pain (*r* = −0.19, *P* = 0.02), pain catastrophizing (*r* = −0.235, *P* = 0.013), and recreational drug consumption (cannabis, cocaine, LSD, etc.; *r* = −0.26, *P* = 0.001). However, when isolating groups, reduction in opioid consumption was predicted by MEQ at hospital discharge for opioid-naïve patients (*r* = 0.55, *P* = 0.001; Figure 2) but not opioid-experienced patients (*r* = −0.12, *P* > 0.05).

Furthermore, the reduction in MEQ from discharge to the final TPS visit for opioid-experienced patients was predicted by less pain catastrophizing (*r* = −0.32, *P* = 0.012), lower levels of neuropathic pain as measured by the SF-MPQ (*r* = −0.265, *P* = 0.04), and a history of recreational drug use (*r* = −0.265, *P* = 0.001).

### Pain and physical function

**Figure 3.**
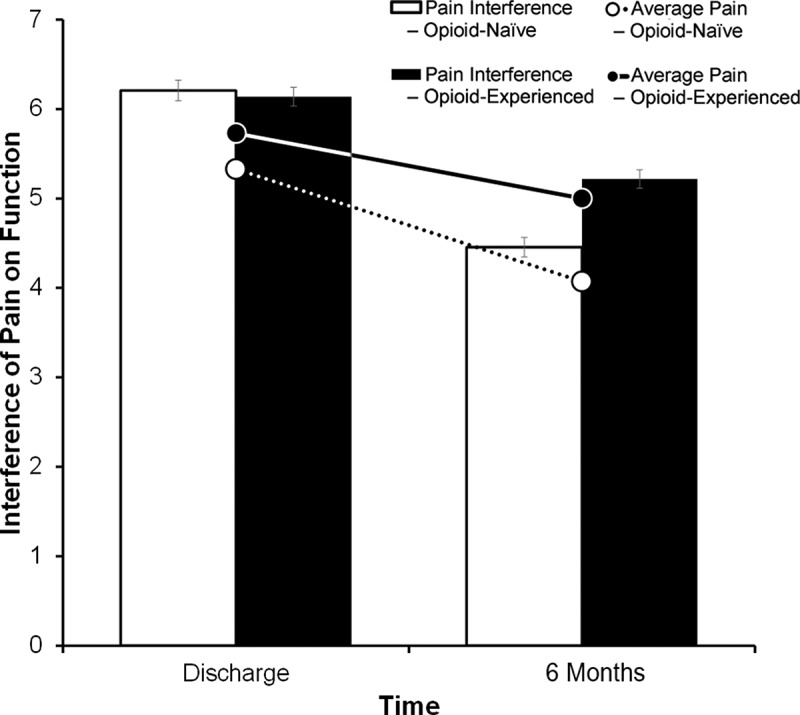
Graph of reductions in NRS pain intensity (lines) and improvements in function from pain interference (bars) of opioid-naïve (white) and opioid-experienced (black) patients. Values are represented as means and standard error bars.

Figure 3 depicts the reductions in pain and improvements in function (as measured by the BPI pain interference score) for the two groups of opioid-naïve and opioid-experienced patients.

Average NRS pain intensity decreased by a mean of 17% for all patients (*n* = 192 with complete questionnaire data). Opioid-naïve patients showed a decrease of 23.6% (5.3 [0.21] to 4.0 [0.20], *n* = 85) and opioid-experienced patients showed a 13% decline (5.7 [0.21] to 5.0 [0.24], *n* = 107) in NRS pain intensity. Using pain at discharge as the covariate, an ANCOVA revealed that mean NRS pain ratings at the final TPS visit differed significantly between the two groups (F = 6.908, *P* = 0.009, η^2^*_p_* = 0.035).

Functional impairment from pain reported by BPI pain interference scores improved by 21% from hospital discharge to the final TPS visit for the total study sample of patients (*N* = 166). ANCOVA, using hospital discharge pain interference scores as the covariate, showed that mean BPI pain interference scores at the final TPS visit were significantly lower (improved) in both opioid-naïve and opioid-experienced groups, with the opioid-naïve group reporting larger improvement than the opioid-experienced group (F = 6.974, *P* = 0.009, η^2^*_p_* = 0.041). Change in pain interference from discharge to final TPS visit amounted to a 28% and 15% improvement, respectively, for the opioid-naïve (*n* = 76) and opioid-experienced (*n* = 90) patients who completed this self-report measure.

### Anxiety and depression

HADS anxiety scores did not show a statistically significant difference within and between groups. Percentage anxiety scores decreased in opioid-naïve patients by a mean of 26% (SD = 45%). Opioid-experienced patients decreased their anxiety scores by a mean of 8% (SD = 57%). HADS depression scores also did not show a statistically significant difference within and between groups. Percentage depression scores decreased in opioid-naïve patients by a mean of 17% (SD = 50%) and in opioid-experienced patients by a mean of 7% (SD = 62%).

## Discussion

This article introduces evaluative findings of the TPS focusing on describing opioid weaning rates (Figure 1), quantifying changes in opioid consumption (Table 3), and evaluating the success of its pain management approach (Figure 3) in patients with complex postsurgical pain. Our findings indicate that a TPS can effectively identify high-risk complex postsurgical pain patients who are struggling with acute postoperative pain. Furthermore, a TPS can allow for gradual tapering from opioids without compromising pain control and psychosocial function, as has been previously described in the detoxification literature.^24^ The results of the present study suggest that intensive follow-up immediately postdischarge, followed by two visits a month for 2 months and monthly for 3–6 months, has a positive impact on opioid consumption and pain outcomes. Because this is a preliminary report documenting the effect size of the TPS, it lacks a formal control group. We have received funding to conduct a multisite randomized controlled trial that includes a control group and will follow this analysis with a pragmatic controlled trial at five institutions to determine the efficacy of the TPS in reducing pain and pain interference as well as in weaning patients from opioids.

There are important highlights to our analysis that deserve further comment. Opioid-naïve patients received an average of 106.7 mg/day of oral morphine (Table 3), well over the 90 MEQ/day limit recommended by 2017 Canadian Opioid Guidelines.^25,26^ We cannot say with certainty that this dose was the minimum necessary dose to adequately control each patient’s pain because many of these patients were discharged from hospital with prescriptions written by surgeons before they became involved with the TPS. A recent report from the province of Ontario, Canada, included statistics showing that 275,778 opioid-naïve patients were discharged from hospital following surgery with 21,297 (8%) on greater than 90 mg of morphine as their starting dose.^27^ Taken together, these data speak to the complexity of managing postsurgical pain and identify a high-risk group that receives high doses of opioids at hospital discharge. These results further highlight the importance of institutional and perioperative oversight and the need to develop multidisciplinary transitional pain services to help patients maximize pain relief and quality of life with the lowest possible dose of opioid.

Until recently, few places in the world have had transitional pain services (acute pain follow-up clinics; European/Scandinavian nomenclature) that aim to (1) prevent the transition of acute pain to chronic postsurgical pain and (2) help patients return to baseline levels of opioid medications or, if possible, wean to zero. It is clear that a proportion of patients cannot be weaned from long-term opioid therapy with traditional means because they have developed chronic postsurgical pain that is effectively managed by their opioid. Doing so with traditional means often involves uncontrolled pain, a return to problematic opioid consumption, and impaired psychosocial functioning. Comparable data on opioid weaning rates in surgical patients are sparse. However, two studies report success with preoperative opioid weaning programs in patients scheduled for spine surgery^28^ and total joint arthroplasty.^29^ In a small pilot study of five patients, an interdisciplinary preoperative weaning program resulted in a reduction in mean daily opioid use from 238 mg (SD = 226.9) before surgery to 139.1 mg (SD = 84.0) one month after surgery for spine surgery patients. Pain interference scores also decreased over time.^28^ A retrospective matched cohort study of patients scheduled for total joint arthroplasty reported that patients who were successfully weaned before surgery by 50% of their preoperative opioid dose showed greater improvements in disease-specific and general health outcome measures than patients who did not wean preoperatively and outcomes comparable to those of patients who preoperatively were opioid naïve.^29^ A multidisciplinary program similar to the TPS reported that 54% and 32% of 200 patients were discharged from hospital after surgery on weak and strong opioids, respectively.^30^ At the end of the outpatient treatment component of the program lasting a median of 3 months, 20% and 6% of patients were reported to be taking weak and strong opioids, respectively. This represents a drop from 171 patients using opioids at hospital discharge to 50 (70.7% decrease) at the end of the outpatient clinic treatment; however, data on preoperative opioid use and daily doses were not reported, making it difficult to compare these data with the present results.^30^ With the TPS, 35 (26%) of our opioid-experienced patients who were taking long-term opioids prior to surgery were able to wean from opioid-based analgesics once engaged in our program. Long-term endpoints including relapse rates are an ongoing part of the development of the TPS and will be included in future program evaluations.

The present study demonstrates that, in our sample of opioid-naïve patients, greater efforts should be taken to maintain the lowest effective MEQ possible while in hospital because it is a strong predictor for the likelihood of weaning long term (Figure 2). To achieve this, multimodal analgesia should be employed with adjunctive medications in order to minimize the MEQ dose. This should be done early and appropriately, particularly in patients who are describing acute neuropathic pain features, which has been previously shown to predict the development of chronic postsurgical pain.^31^ In the more challenging opioid-experienced patient cohort, we demonstrated a greater wean with the use of the behavioral ACT intervention provided by the TPS. A recent study demonstrated that patients referred to an ACT intervention demonstrated greater reductions in opioid use and pain interference and showed reductions in depressed mood by the end of treatment.^16^ Given the success of this subgroup of difficult patients, we are developing a model for postoperative weaning that integrates the ACT paradigm into a pragmatic randomized controlled trial.

Another notable finding is that 21 of the 26 opioid-experienced patients who increased opioid consumption after discharge were male (Table 3, Figure 1). This supports previously published studies on the predominance of males in opioid-experienced populations. A study by Rahman and colleagues focused on thousands of chronic pain patients utilizing the Manage My Pain chronic pain mobile application. It demonstrated that males tend to self-report higher doses for opioid medications than their female counterparts.^32^ Another study looking at the Ontario population demonstrated that males were at higher risk of dying of an opioid overdose than females within the general population.^33^ Thus, special attention should be provided to younger males being discharged on higher doses of opioid medications postoperatively.

One clear limitation is the need to develop a system for follow-up once patients are discharged from our TPS to determine whether patients continue to be opioid free. We are currently attempting to link patient data to our provincial health care databases to determine whether prescription outcomes change in the long term. Related to this is the limitation of data extending to only 6 months after surgery on average. Anecdotally, there remains a subpopulation within the TPS that extends well beyond our targets for discharge. Therefore, some of the conclusions drawn from this data are based on patients who have continued with the TPS beyond 6 months because they tend to have more severe psychosocial functioning limitations and may skew these outcomes. More community resource allocation is needed for chronic pain patients^34,35^ in order to transfer the needs of these complex patients back to the primary care setting. If this can be done efficiently and in a timely manner, we can circumvent a capacity bottle neck that prevents access to other patients who require transitional pain services, approximately 5%−15% of the surgical volume of any surgical institution.^12,13^

Lastly, the lack of randomization and a non-TPS control group limits our ability to draw clear conclusions with respect to our intervention when compared to no intervention, standard of care, or other opioid-weaning strategies. Given the success of our current program with respect to opioid weaning following major surgery—that is, 45% of opioid-naïve and 26% opioid-experienced completely weaned and 80% and 61% respectively weaned to less than half their baseline dose (Table 3, Figure 1)—we have been funded to move forward with a multisite randomized controlled trial focused on replicating this program in urban and rural settings in the province of Ontario, Canada. This will provide conclusive evidence regarding the effectiveness of the TPS as other institutions across the country begin to implement similar programs.

In conclusion, close monitoring of patients in the acute postoperative setting and timely identification/intervention of the ~15% of surgical patients who are not recovering appropriately while in hospital and being discharged on high-dose opioids should become a priority within perioperative care over the coming years. We have previously published on the cost associated with the development of chronic postsurgical pain as a consequence of the top ten priority surgeries in Canada to be an estimated 1.8 billion dollars annually to the Canadian economy.^14^ The development, staff, and overview of the TPS has been previously described.^17,18^ In addition, we believe that a TPS can function effectively with as few as three professionals (anesthesiologist, psychologist or other mental health care professional, and nurse or nurse practitioner). The time has come for perioperative physicians and institutions to face the potential negative consequences associated with life-saving surgery and create supports for the complex postsurgical pain patient. Further research will elucidate whether long-term investment into transitional pain clinics should be made a priority and whether the involvement of e-technology in this process might enhance the ability for widespread availability and uptake.
